# Clinico-Epidemiological Profile of Leptospirosis Among Patients Attending a Tertiary Care Center in Rajasthan

**DOI:** 10.7759/cureus.114221

**Published:** 2026-08-09

**Authors:** Rajesh K Sharma, Sakshee Gupta, Megha Sharma, Pratibha Sharma, Himanshu Sharma, Simmi Tiwari, Ajit Shewale, Jitesh Kuwatada, Bharti Malhotra

**Affiliations:** 1 Department of Microbiology, Sawai Man Singh (SMS) Medical College and Hospital, Jaipur, IND; 2 Centre for One Health, National Center for Disease Control, New Delhi, IND; 3 Center for One Health, National Centre for Disease Control, New Delhi, IND

**Keywords:** clinical profile, diagnostic trends, epidemiology, igm capture enzyme-linked immunosorbent assay (elisa), leptospirosis

## Abstract

Background and objective

Leptospirosis is a globally prevalent zoonotic disease with a differential diagnosis of acute febrile illness that is significantly underreported in Rajasthan, India. This study aimed to assess the seropositivity for *Leptospira *in patients attending a tertiary care center in Rajasthan and study their clinico-epidemiological profile.

Material and methods

A cross-sectional study was conducted between July 2019 and June 2022 at the Advanced Research Laboratory, Department of Microbiology, Sawai Man Singh (SMS) Medical College and Hospital, Jaipur, Rajasthan, India. Serum samples from 2,996 patients presenting with undifferentiated febrile illness/pyrexia of unknown origin (UFI/PUO) were tested for *Leptospira* IgM antibodies using an IgM capture enzyme-linked immunosorbent assay (ELISA). A total of 290 patients with a duration of illness of less than five days underwent *Leptospira* testing by polymerase chain reaction (PCR). Demographic characteristics, clinical features, exposure history, and duration of animal contact were obtained from the specimen requisition forms. Data were analyzed using appropriate statistical methods.

Results

A seropositivity of 5.01% (150/2,996) for *Leptospira *was found in the present study, with a male predominance of 58.67% (88/150) and 41.33% (62/150) female among positive cases. All samples (290) tested negative by PCR.

The highest positivity was observed in the age group of 19-30 years. Highest positivity was observed during the monsoon and post-monsoon periods and winter (July-December, peak December) in Rajasthan. Seasonal analysis revealed that December consistently showed high positivity (10.70%), followed by July (8.53%), October (8.0%), and February (6.07%). However, the highest positivity was seen in November 2020 (36.36%) and December 2020 (20%).

All 150 patients reported symptoms like fever and myalgia, whereas 76% (114/150) presented with headaches. Prolonged contact with animals and lack of awareness about zoonotic diseases were identified as major risk factors contributing to disease transmission in Rajasthan.

Conclusion

Leptospirosis is an underdiagnosed cause of acute febrile illness in Rajasthan, with animal exposure being a significant risk factor in our setting, particularly during the monsoon and post-monsoon seasons. Enhanced diagnostic facilities, increased awareness among clinicians, and targeted public health interventions are crucial to reducing associated morbidity and mortality.

## Introduction

Leptospirosis is a pervasive zoonotic disease caused by pathogenic species of *Leptospira *and has been documented in India since 1931 [1]. Recognized as the most widespread zoonotic disease, it is particularly prevalent in tropical and low-income regions.

Transmission to humans typically occurs through direct or indirect contact with soil or water contaminated by the urine of infected animals, with rodents, dogs, cattle, and pigs serving as primary reservoir hosts.

The prevalence of leptospirosis has increasingly been reported in the last few years from various parts of India, especially after heavy rains and from areas of unplanned urbanization and in persons exposed to contact with animals [1].

A major challenge in the diagnosis of leptospirosis is that the signs and symptoms are similar to other tropical infections like malaria, typhoid, dengue, chikungunya, rickettsial infections, viral hepatitis, etc. A confirmed diagnosis can be provided by specific laboratory investigation only; however, the limitation is that the facilities are not available in most hospitals. As a result, the disease goes unreported and misdiagnosed [1, 2]. The situation of overlapping symptoms, low clinical suspicion, and lack of diagnostic facilities has led to underreporting of the disease; consequently, patients do not receive specific therapy, leading to increased severity of illness and higher mortality [2]. As a result of these compounding factors, it’s an important public health issue that should be addressed. There is limited data on the prevalence of leptospirosis in Rajasthan [2,3].

We carried out the present prospective, cross-sectional study to evaluate the clinico-epidemiological profile of leptospirosis among patients presenting with undifferentiated febrile illness (UFI)/pyrexia of unknown origin (PUO) at a tertiary care center in Rajasthan from July 2019 to June 2022.

## Materials and methods

Study design, setting, and duration

This study was a prospective cross-sectional study carried out at Sawai Man Singh (SMS) Medical College and Hospital and associated hospitals in Jaipur, Rajasthan, from July 2019 to June 2022. The study received ethical approval from the Institutional Ethics Committee (approval number 755 MC/EC/2019), and written informed consent was obtained from all participating patients or their family members before enrollment.

Patient enrollment and inclusion criteria

The study enrolled 2,996 patients as per the random stratified sampling method, presenting with UFI or PUO from both outpatient and inpatient departments. Negative investigation reports of various infectious diseases were used to select these patients, and no follow-up was done. 

Participant flow: Inclusion and exclusion criteria

A total of 6,010 febrile patients attending SMS Medical College and Hospital and attached hospitals were shortlisted between July 2019 and June 2022. Samples were tested for dengue IgM, NS1 antigen, and chikungunya IgM. Those negative for dengue and chikungunya (1,256) were excluded; the rest were further excluded based on non-resident status in Rajasthan (734) and those not falling in UFI/PUO (185). Patients positive for malaria, typhoid, COVID-19, and influenza were also excluded (839). Representative samples (40-100 samples per district) were randomly selected (2,996) and tested for *Leptospira*. 

Case definitions of UFI and PUO were adapted from Harrison's Principles of Internal Medicine [4]. UFI is defined as a fever lasting less than two weeks, while PUO is defined as a prolonged fever >3 weeks that remains undiagnosed after thorough investigation. Patients not falling in these categories were excluded from the study. The patients who refused consent or provided inadequate samples were also excluded. There were no pre-defined criteria for age and antibiotic use in the present study. 

History and epidemiological data, including age, sex, address, occupation, duration of contact with animals (buffalo, cattle, and rodents); prolonged contact with water (swimming)/meat/vegetables; past fever (nature and duration); and other clinical complaints, were collected from all study participants by trained project staff in the validated questionnaire/requisition forms (Appendix A).

Minimum sample size calculated by the formula: $n = \frac{z^2 p q}{d^2}$, where p = prevalence rate; q = (100-P); d = permissible error margin; and z = constant at 99% C.I. z = 6.66. Taking *Leptospira *seroprevalence to be 6.47% as per Agarwal et al. [5], minimum sample size required for the study was found to be 2,978 using the formula, where prevalence (p): 6.47% (0.0647), value of q = 1-p = 0.935, error margin (d): 3% (0.03), and value of z at 99.99% confidence level (Z): 6.66. Hence, a total of 2,996 samples were recruited for the present study by the random sampling method. A high confidence interval of 99.9% was chosen to get samples from the maximum number of districts of Rajasthan. 

Serological methods

A 3-5 mL venous blood sample was collected from each patient and assigned a unique identification number. Serum was separated by centrifugation and divided into two aliquots. One aliquot was stored at −20°C as a backup sample, while the other was used for serological testing. The samples were tested for Dengue NS1 antigen using an enzyme-linked immunosorbent assay (ELISA) (MICROLISA Dengue NS1 Antigen ELISA, Catalog No. EOA031122, J. Mitra & Co. Pvt. Ltd., New Delhi, India); dengue IgM antibodies using the Dengue IgM Capture ELISA (ICMR-National Institute of Virology (NIV), Pune, Maharashtra, India); chikungunya IgM antibodies using ELISA (Catalog No. IR053096, J. Mitra & Co. Pvt. Ltd., New Delhi, Delhi, India); and anti-*Leptospira *IgM antibodies using the Panbio™ Leptospira IgM ELISA (Catalog No. 02PE10, Abbott Diagnostics, Brisbane, Queensland, Australia). According to the manufacturer's instructions, the Panbio™ *Leptospira* IgM ELISA has a reported sensitivity of 96.5%, specificity of 98.5%, coefficient of variation ranging from 3.4% to 10.3%, and no reported cross-reactivity with other infectious agents [6]. Absorbance was measured at 450 nm using an ELISA microplate reader. The calibrator factor (CF) provided in the manufacturer's kit insert was used to calculate the assay cut-off value as calibrator optical density (OD) × CF. Results for anti-*Leptospira *IgM antibodies obtained by indirect ELISA were included in the present study. To exclude false-positive results due to rheumatoid factor interference, positive samples were further tested using a rheumatoid factor latex agglutination test (Catalog No. RF/00, Precision Bio Med Pvt. Ltd., Chomu, Jaipur, Rajasthan, India).

Molecular methods

DNA was extracted from whole blood using the QIAamp DNA Blood Mini Kit (QIAGEN GmbH, Hilden, North Rhine-Westphalia, Germany) according to the manufacturer's instructions and subsequently tested by real-time polymerase chain reaction (real-time PCR). Multiplex real-time PCR was performed using the AgPath-ID™ One-Step RT-PCR Kit (Applied Biosystems™, Thermo Fisher Scientific, Foster City, CA, USA) along with primers and probes specific for the detection of *Leptospira *and the endogenous internal control RNase P, as previously described [7]. Briefly, the reaction master mix consisted of 12.5 µL of 2× RT-PCR buffer, 1 µL of RT-PCR enzyme mix, and 1 µL each of the primer-probe mix (PPM) for *Leptospira *and RNase P. The final reaction volume was adjusted to 20 µL with nuclease-free water. Subsequently, 5 µL of extracted DNA was added to the 20 µL reaction mixture to obtain a final reaction volume of 25 µL. Amplification was performed on a QuantStudio™ 5 Real-Time PCR System (Applied Biosystems™, Thermo Fisher Scientific, Foster City, CA, USA). The thermocycling conditions consisted of an initial denaturation at 95°C for 10 minutes, followed by 45 cycles of denaturation at 95°C for 15 seconds and annealing/extension at 55°C for 30 seconds. A positive control and a no-template negative control were included in each run to ensure assay performance and monitor contamination.

Statistical analysis

For the study, descriptive statistics such as categorical variables and continuous variables were summarized within the study population. Variables with normal distribution were expressed as the mean and standard deviation. The chi-square test and Fisher's exact test were applied using IBM SPSS Statistics version 21.0 (IBM Corporation, Armonk, NY, USA). The Shapiro-Wilk test was used to verify normal distribution of patient data; a p-value of less than 0.05 indicated statistical significance. *Leptospira*-negative patients were used as the comparator/control group. 

## Results

Among the 2,996 patients enrolled, 1,693 were categorized as UFI (duration of illness <2 weeks) and 1,303 as PUO (duration of illness >3 weeks). Seropositivity by *Leptospira *IgM ELISA was 7.68% (130/1,693) for UFI and 1.53% (20/1,303) for PUO patients, respectively. Overall, seropositivity of leptospirosis was found to be 5.01% (150/2,996) in the present study. All 290 samples/cases (with < 5 days of illness) tested for *Leptospira *PCR were found to be negative.

The study observed a male predominance with 58.67% (88/150) and 41.33% (62/150) females among positive cases. The majority of cases were found in the 19-30 year age group (43.6%), followed by 31-40 years (17.96%) (Table 1). Urban residents accounted for 52.7% of the cases, while rural residents comprised 47.3%, with no statistically significant difference between these groups.

**Table 1:** Age-wise distribution of Leptospira seropositive cases from Rajasthan *Fisher’s exact test was applied. p-value ≤0.05 was considered significant. N=number.

Age group (years)	Total positive/Total tested (%)	Age-wise distribution of *Leptospira *seropositive cases from 2019-2022		
		Male (%)	Female (%)	p-value
0-10	8/145 (5.51)	7 (4.83)	1 (0.69)	0.14
11-18	18/300 (6.00)	10 (3.33)	8 (2.67)	0.47
19-30	61/1307 (4.67)	33 (2.52)	28 (2.14)	0.28
31-50	44/872 (5.05)	26 (2.98)	18 (2.06)	0.55
>50	19/372 (5.11)	12 (3.23)	7 (1.88)	0.44
Total	150/2996 (5.01)	88 (2.94)	62 (2.07)	

Out of a total of 2,996 samples tested, 150 samples (5.01%) were seropositive for *Leptospira*. Male patients in the pediatric group (0-18 years of age) had a positivity of 11.33% (17/150), and female patients had a positivity of 6% (9/150). Male patients aged 19-50 years had a notably high positivity rate of 39.33% (59/150), and female patients showed 30.67% (46/150). The elderly male population (>50 years group) had a positivity of 8% (12/150), while for the female population, it was 4.67% (7/150). However, no statistically significant differences in seropositivity were found between male and female patients across all years from 2019 to 2022 and age groups.

**Figure 1:**
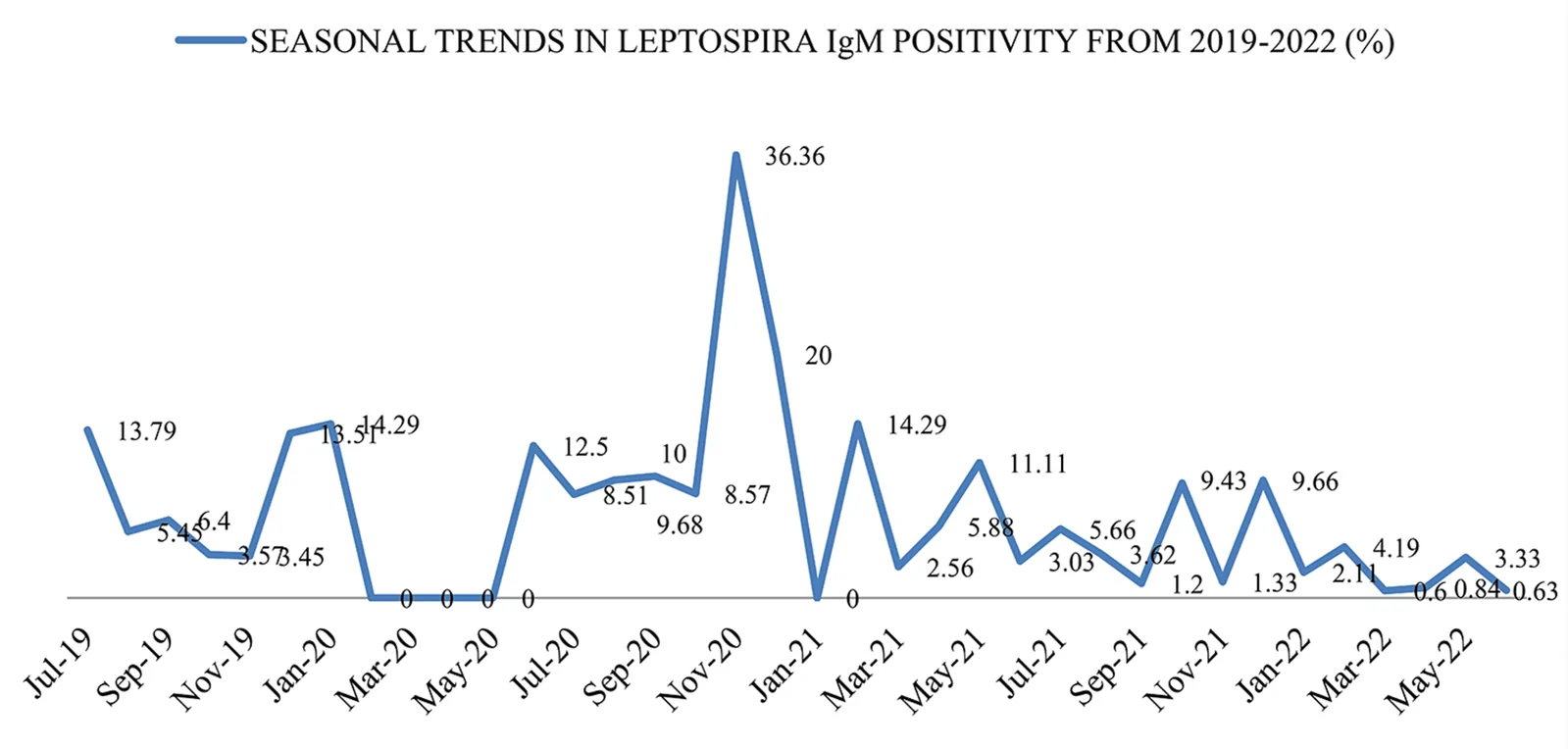
Seasonal distribution of seropositive pyrexia of unknown origin (Leptospira) patients from 2019-2022. The data are in percentages (%).

Figure 1 shows the seasonal distribution of seropositive PUO (*Leptospira*) patients from 2019 to 2022. Month-wise, the highest positivity was observed in November 2020 (36.36%), December 2020 (20%), January 2020 & February 2021 (14.29% each), July 2019 (13.79%), December 2019 (13.51%), July 2020 (12.5%), etc. A clear seasonal pattern was observed, with markedly highest positivity in cases during the monsoon, post-monsoon, and winter periods (July-December). Seasonal analysis revealed that December consistently showed the highest positivity (10.70%), followed by July (8.53%), October (8.0%), and February (6.07%). indicating a seasonal trough during the hot and dry period. February (p <= .016), June (p = 0.025), September (p = 0.032), and November (p < 0.001) showed a significantly higher proportion of positive cases compared to other months.

Table 2 shows the clinical characteristics in *Leptospirosis*-positive cases. Fever and myalgia were reported in 100% (150/150) of the cases, followed by headache in 76% (114/150) and arthralgia in 56.67% (85/150). Severe symptoms such as jaundice occurred in 54 (36%), renal syndrome in 38 (25.33%), and meningeal signs in 33 (22%) cases. Less common features included hemoptysis (7 (4.67%)) and rash (6 (4%)). No statistically significant differences were observed (p>0.05).

**Table 2:** Clinical characteristics of Leptospirosis-positive cases # Many patients had more than one symptom. ‡Co-morbidities total: (5.3%, 8/150) like hypertension (1.3%, 2/150), diabetes (0.67%, 1/150), ischemic heart disease (0.67%, 1/150), atrial fibrillation (0.67%, 1/150), chronic obstructive pulmonary disease (1.3%, 2/150), and chronic kidney disease (0.67%, 1/150) were present in study participants. *Fisher’s exact test was applied. p-value ≤0.05 was considered significant.

Clinical characteristics	Total *Leptospira-*positive cases from 2019-2022=150	
	Total; Male/Female n (%)	p-value
Fever	150 (100)	1.00
Rash	6 (4.00)	0.06
Headache	114 (76.00)	1.00
Myalgia	150 (100)	1.00
Chills and rigor	20 (13.33)	0.58
Arthralgia	85 (56.67)	0.51
Nausea & vomiting	46 (30.67)	0.10
Diarrhoea	38 (25.33)	0.65
Conjunctival suffusion	57 (38.00)	0.89
Cough	30 (20.00)	1.00
Hemoptysis	7 (4.67)	0.44
Meningeal signs	33 (22.00)	0.28
Jaundice	54 (36.00)	0.23
Renal syndrome	38 (25.33)	0.82

The symptom onset timeline showed fever beginning at day 0 (mean 5.3 ± 1.3 days), followed by headache and myalgia within 1-1.5 days, arthralgia and gastrointestinal symptoms by day 2.5-3.5, conjunctival suffusion by day 3.5, jaundice by day 6.5, and renal or meningeal signs appearing between days 9 and 11.

Table 3 shows the history of animal contact in *Leptospirosis*-positive cases. Animal contact was reported in 77.33% of positive cases, with buffalo (39.33%) and cattle (cows, goats, and sheep) (5.33%-23.33%) as predominant exposures. While none of the cases reported camel contact, 2.67% of *Leptospira*-positive cases reported contact with household rodents and 4.6% had a history of prolonged contact with water (swimming).

**Table 3:** History of animal contact in Leptospirosis-positive cases ^#^Many patients had exposure to more than one type of animal.

Type of animal handling	History of animal contact in *Leptospira-*positive cases (150) from 2019-2022
	N (%)
Buffalo	59 (39.33)
Cow	35 (23.33)
Goat	10 (6.67)
Sheep	8 (5.33)
Camel	0 (0.00)
Rodents	4 (2.67)
Animal handling expert	1 (0.67)
Total patients with animal contact^#^	116 (77.33)
No animal contact	70 (46.67)
History of prolonged contact with water (swimming)	7 (4.67)

**Figure 2:**
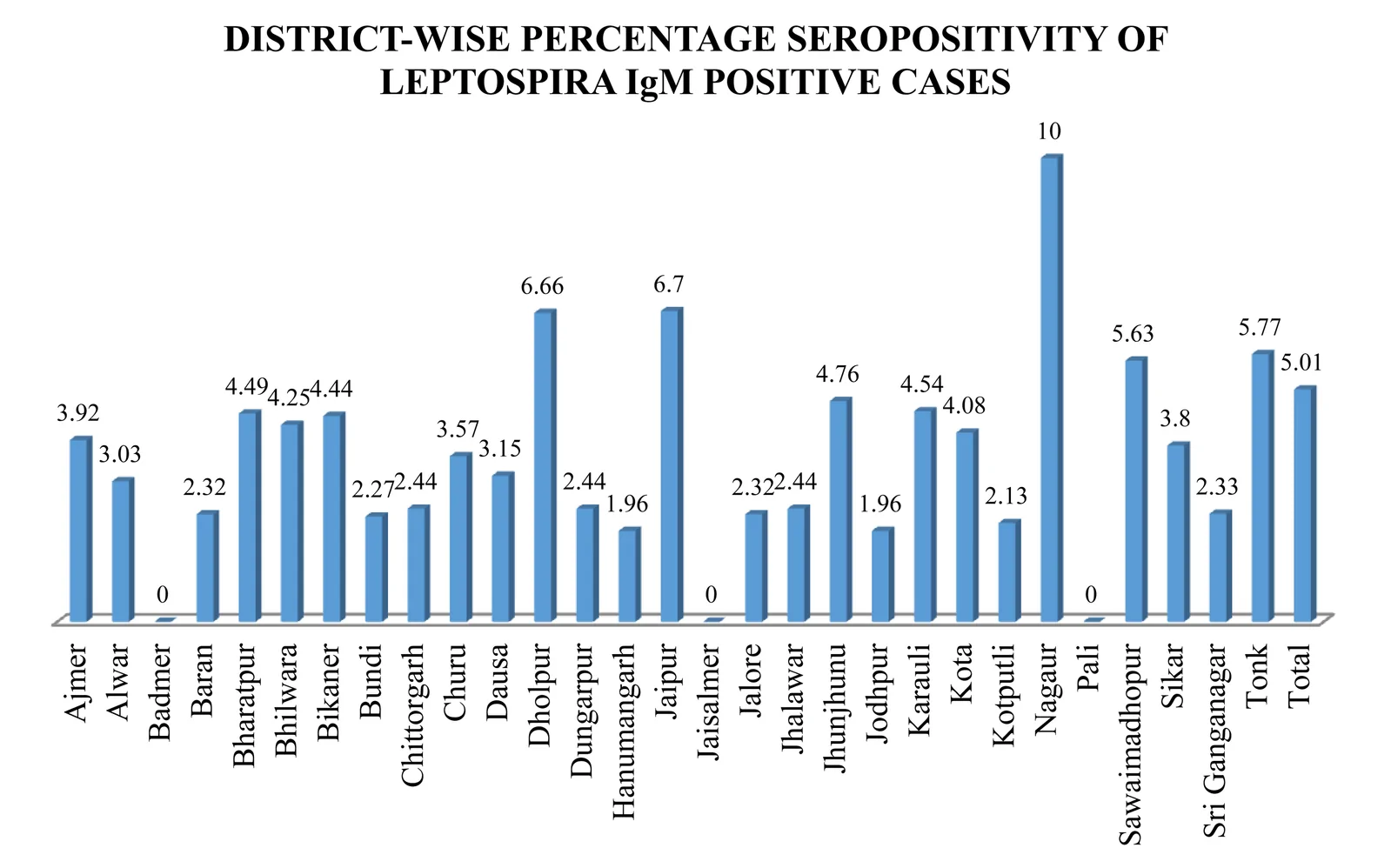
District-wise percent seropositivity of Leptospira IgM-positive cases The data are in percentages (%).

Figure 2 shows the district-wise percent seropositivity of *Leptospira *IgM-positive cases. Highest seropositivity was observed in Nagaur (5/50, 10%), followed by Jaipur (87/1,298, 6.70%), Dholpur (4/60, 6.66%), Tonk (3/52, 5.77%), Sawai Madhopur (8/142, 5.63%), Jhunjhunu (3/63, 4.76%), and Bharatpur (4/89, 4.49%). Three districts (Barmer, Jaisalmer, and Pali) did not report any positive cases.

District-wise distribution showed Nagaur as a significant hotspot, accounting for 3.3% (10/150) of cases, with other affected districts including Jaipur, Dholpur, Tonk, and Sawaimadhopur.

## Discussion

A seropositivity of 5.01% for leptospirosis found in our study closely mirrors observations by Patel et al. (77/1611, 4.78%) [8] from North India. They studied a cohort of PUO patients, among which 7.11% were IgM ELISA-positive for leptospirosis.

Kumar et al. [9] studied 200 suspected leptospirosis patients and found leptospirosis seropositivity to be 24.5% (49/200) at Ranchi, Jharkhand, India.

A descriptive cross-sectional study was done by Sharma et al. [10] on serosurveillance of leptospirosis among veterinary personnel in Sikkim, India, and found 15.9% (49/308) seropositivity for *Leptospira*.

The male predominance observed in our study (58.67%; 88/150) is consistent with previous Indian studies [11], which have reported a higher proportion of male patients, largely attributed to greater occupational exposure to farming, livestock handling, and other outdoor activities. Kembhavi et al. [12] observed that males constituted 73% of urban and 62.4% of rural leptospirosis cases in Maharashtra, reflecting greater occupational and environmental exposure to contaminated water and livestock.

Our finding that young adults (19-30 years, 43.6%) and middle-aged persons (31-40 years, 17.96%) are most affected is consistent with the age distribution described by Sethi et al. [11] and Kembhavi et al. [12], who found similar vulnerability among working-age adults engaged in high-risk activities.

While urban cases represented a slight majority (52.7%) in our data, Bhardwaj et al identified flooding-related exposure as a major determinant of leptospirosis and recommended intensified rodent control and health education [13]. Mudur et al .have observed seasonal surges and high endemicity during monsoon season in municipal wards of Mumbai [14].

A recent cross-sectional study from Gujarat by Mevada et al. reported an overall seropositivity of 46.3% among high-risk groups. The highest seropositivity was observed among poultry farm workers (56.6%) and cattle farm workers (54.5%). The authors recommend regular surveillance and preventive measures. [15]. Similarly, Pappachan et al. from Kerala also reported outbreaks of *Leptospira *during the monsoon period in the rural population working in paddy fields and livestock handlers [16].

In our study, we observed higher positivity in the monsoon and post-monsoon periods, which is similar to that reported by Kembhavi et al. [12] from Maharashtra, who observed a surge in leptospirosis cases during the monsoon and post-monsoon, linking it to heavy rainfall, flooding, and factors affecting the burrows of rodents. Similarly, a study by Robertson et al. [17] from the neighbouring country of Sri Lanka also observed higher positivity and increased incidence of outbreaks during monsoon, particularly after heavy rainfall. However, Gupta et al. [18] reported cases even during non-monsoon time in areas where good hygiene and drainage were in place. In our study, a surge (36.36%) of cases in 2020 could be due to very heavy rainfall leading to waterlogging in different areas, as a result affecting rodent populations and sanitary conditions.

In our study, all the patients had fever (100%), myalgia (100%), arthralgia (56.67%), jaundice (36.00%), renal syndrome (25.33%), and meningeal signs (22.00%). Similar findings were reported by Kumar et al. [9], who observed fever (98%), myalgia and headache (72%), and jaundice (19%), which appeared seven days after onset of fever. Whereas a study from Kerala [19] reported that 54% of their patients had jaundice and renal syndrome, which occurred six to eight days after disease onset, a study from Brazil reported that a few cases developed jaundice and renal complications in three to five days only, which could either be due to host factors or the pathogen [20]. Rajapakse et al. from Sri Lanka also reported that a few cases had more severe illness and rapid progression [21].

In our study, we observed a history of animal contact as a major risk factor (77.33%), mainly due to buffalo and cattle. Similar findings have also been reported by Patil et al., who found cattle rearing and farm contamination to be the main source of transmission of disease [22]. A study from South India reported goats and sheep to be the main reservoirs, with 44% and 38% of cases, respectively [23]. Another study reported that in many countries camels have contributed to disease transmission, but not so in India, as also observed in the present study [24].

We carried out IgM ELISA for diagnosis of *Leptospira *in our study, which has been reported to have >90% sensitivity; additional testing by PCR is also recommended to identify early-stage infections [22,25,26].

As reported by the systematic review and meta-analysis by Gupta et al., leptospirosis cases in India were predominantly found clustered in the coastal states of Gujarat, Tamil Nadu, Maharashtra, Kerala, Karnataka, and the Andaman and Nicobar Islands, whereas epidemiological evidence from Rajasthan remains limited [18]. Sethi et al. similarly recommended intensive rodent control and sanitation improvements in hotspot districts after demonstrating case clustering patterns following rainfall events [11]. Multi-author reviews emphasized that population awareness campaigns and infrastructure upgrades before and during monsoon seasons remain essential for outbreak mitigation.

Limitations of the study

Testing was performed using only the IgM serological kit. It is recommended to test both IgM and IgG to elucidate a clearer epidemiological picture of the exposed population and risk factors associated with leptospirosis. Moreover, culture was not performed, which is the gold standard method for diagnosis. Microscopic agglutination test (MAT) for serovar identification was not performed. PCR was performed only in a small number of cases. Samples were not collected directly from all districts of the state. Instead, representative patients presenting to SMS Hospital, Jaipur, from different districts were tested to estimate the prevalence of leptospirosis from various districts of Rajasthan. This may have caused selection bias, as higher numbers were from Jaipur. Moreover, as samples were selected from patients who were negative for common infections, we may have missed cases having coinfection with two or more pathogens. 

## Conclusions

Leptospirosis is an underdiagnosed zoonotic infection, as we are not regularly testing for these pathogens in the state. Data have emerged that these diseases are prevalent in our state and should be tested for in PUO/UFI cases. It's important that high clinical suspicion be maintained for these well-known old diseases, which seem to be emerging/re-emerging in our state. Prolonged occupational contact with farm/poultry animals poses a significant epidemiological risk of contracting infection, particularly in the post-monsoon season. Strengthened sero-surveillance will aid in early laboratory diagnosis of leptospirosis. This must be coupled with targeted public health measures for controlling this infection.

## Figures and Tables

**Figure 3:**
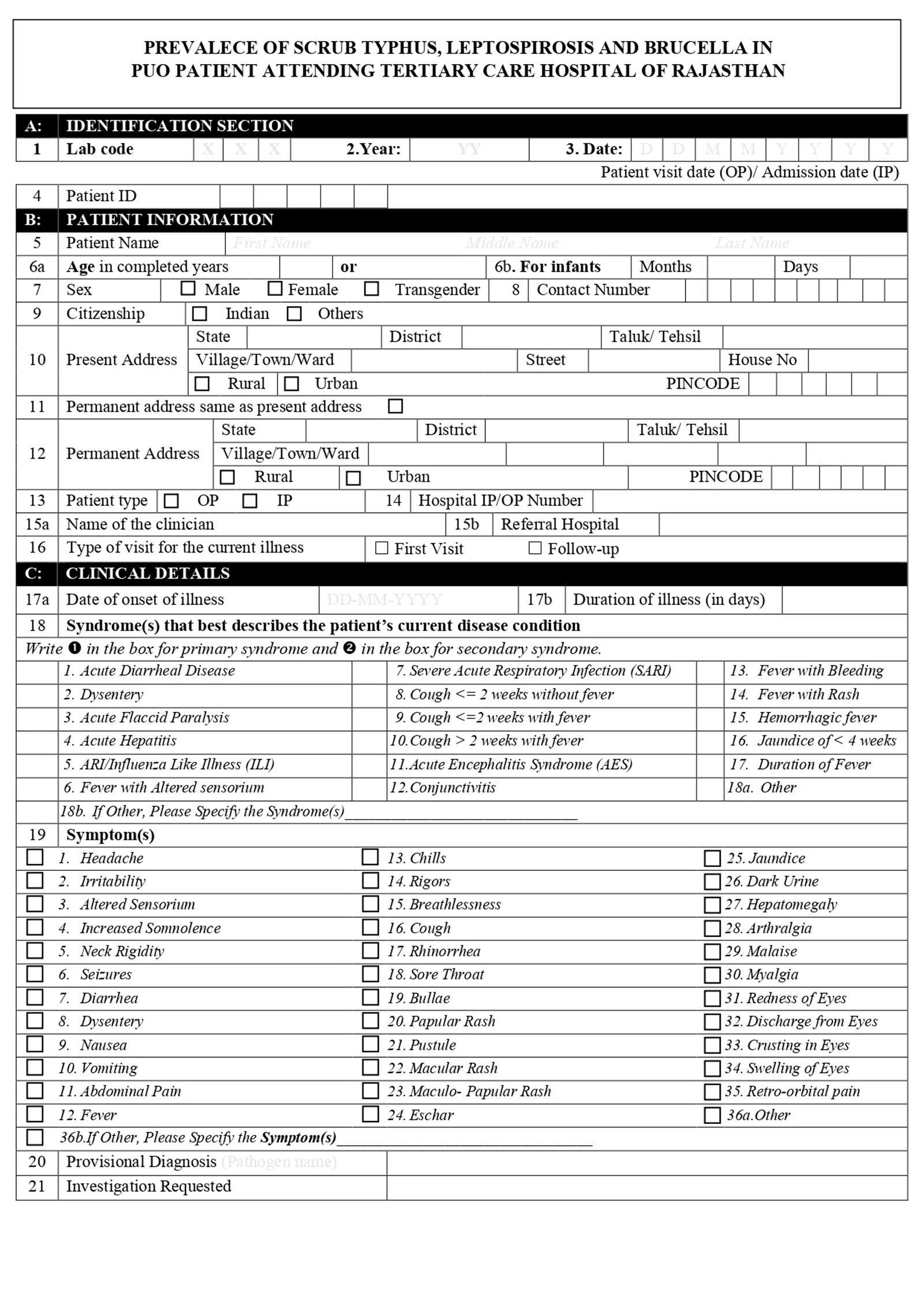
Validated questionnaire: part 1

**Figure 4:**
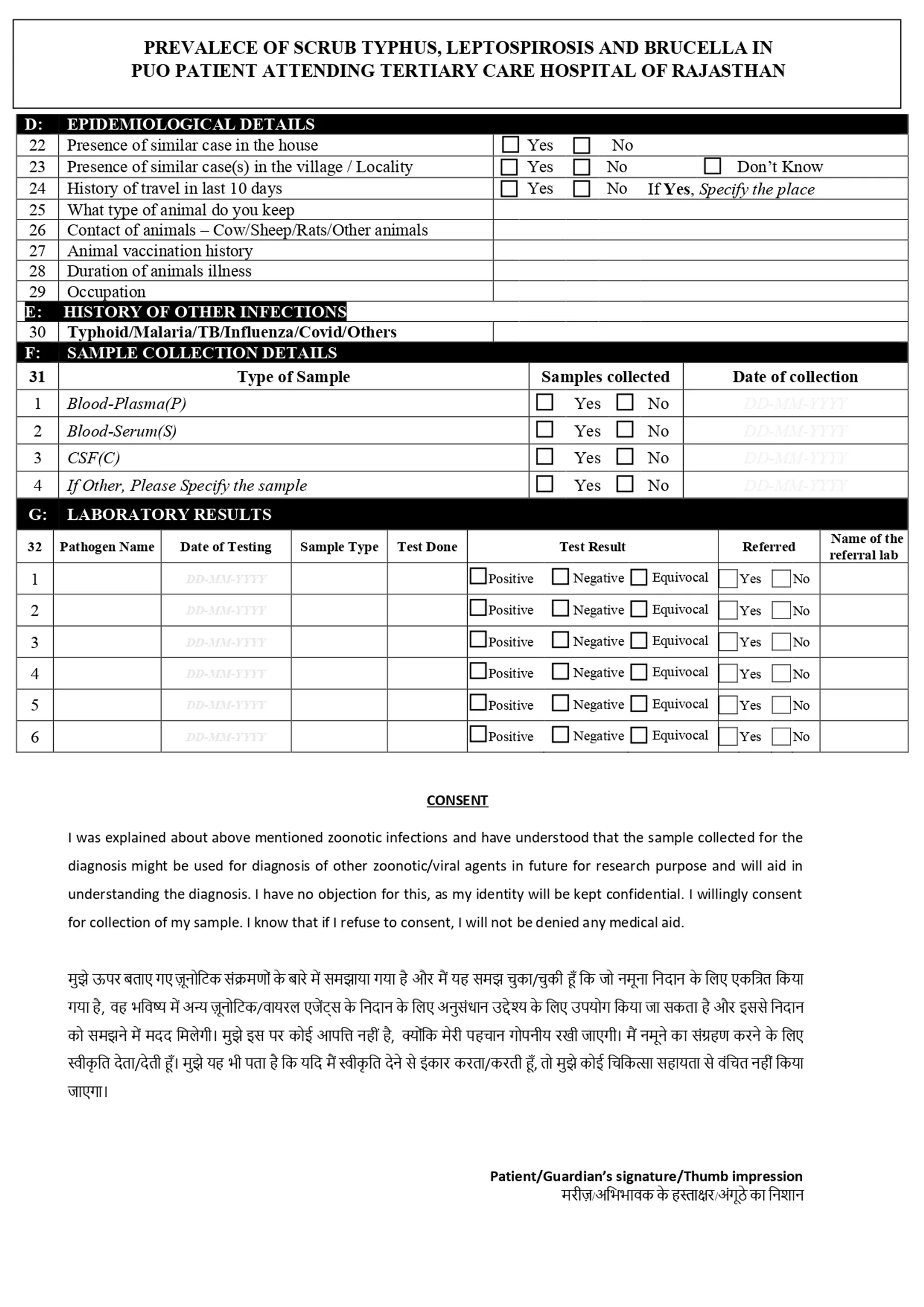
Validated questionnaire: part 2

## Appendices

Appendix A
